# Multidisciplinary Rescue Airway Management in a Self-Inflicted Penetrating Neck Injury: A Case Report

**DOI:** 10.7759/cureus.99111

**Published:** 2025-12-13

**Authors:** Ana Rita Góis, Kateryna Samalyuk, Fátima Costa

**Affiliations:** 1 Anesthesiology, Unidade Local de Saúde de Coimbra, Coimbra, PRT

**Keywords:** airway management, cervical trauma, laryngeal transection, multidisciplinary team, tracheotomy, unconventional intubation

## Abstract

Self-inflicted penetrating neck injury is a rare but highly lethal form of suicide attempt, often resulting in complex injuries to the upper airway and digestive tract. We report a case of a man in his 50s presenting with a complete supraglottic laryngeal and partial pharyngeal transection due to self-inflicted stab wounds. The initial assessment took place in a non-tertiary hospital, and an emergency airway access was achieved via tracheal intubation through the open cervical wound, followed by transfer to a level I trauma center and urgent surgical repair. A multidisciplinary team involving anesthesiology, intensive care, general surgery, and otorhinolaryngology ensured a favorable outcome. This case highlights the need for adaptable airway strategies and multidisciplinary collaboration in the management of traumatic airway disruption.

## Introduction

A rare and highly lethal method of suicide is self-inflicted penetrating neck injury, which may involve transection of the hypopharynx, larynx, trachea, esophagus, and, sometimes, major neck vessels. Penetrating neck trauma, most commonly resulting from stab wounds, should be distinguished from blunt mechanisms such as hanging, strangulation, or self-inflicted blunt trauma. As reviewed by Loss et al., neck injuries - most commonly resulting from stab wounds - account for up to 10% of all trauma cases, with mortality rates reaching as high as 10% [1,2]. In recent years, several European countries have reported an increase in such injuries, particularly among patients with psychiatric disorders or in the context of interpersonal violence [2].

These injuries represent a critical and challenging emergency, as securing the airway and maintaining effective ventilation and oxygenation can often be extremely difficult [3,4]. The neck is traditionally divided into three zones: Zone I (clavicles to cricoid cartilage), Zone II (cricoid to angle of the mandible), and Zone III (angle of the mandible to the base of the skull). The location of the injury significantly influences both diagnostic and therapeutic strategies [1].

A multidisciplinary approach is essential, with airway management as a major concern due to the frequent disruption of normal anatomy and the associated risks of hypoxia, difficult ventilation, and aspiration of blood and/or gastric contents. Other complications, such as severe bleeding and hypovolemic shock, can increase the risk of morbidity and mortality. In such cases, direct intubation, surgical airway access, or fiberoptic-assisted techniques may be necessary, depending on the extent and location of the injury [3-5]. Therefore, it is essential to understand how to perform the initial management of a compromised airway until the patient is stabilized and can be transferred to a hospital capable of securing a definitive airway repair [1].

We report a case of a patient who survived a self-inflicted penetrating neck injury and describe the multidisciplinary management strategy adopted.

## Case presentation

A male patient in his 50s, classified as American Society of Anesthesiologists (ASA) Physical Status I E (emergency) was admitted after a self-inflicted penetrating cervical injury with a knife, resulting in a 15-20 cm transverse anterior cervical wound, between the cricoid cartilage and the angle of the mandible, with complete laryngeal transection (supraglottic), partial pharyngeal transection, subcutaneous emphysema, and a left pneumothorax from additional thoracic stab wounds on the left hemithorax. No major vascular injury was identified, and the cervical injury showed no active bleeding.

**Figure 1:**
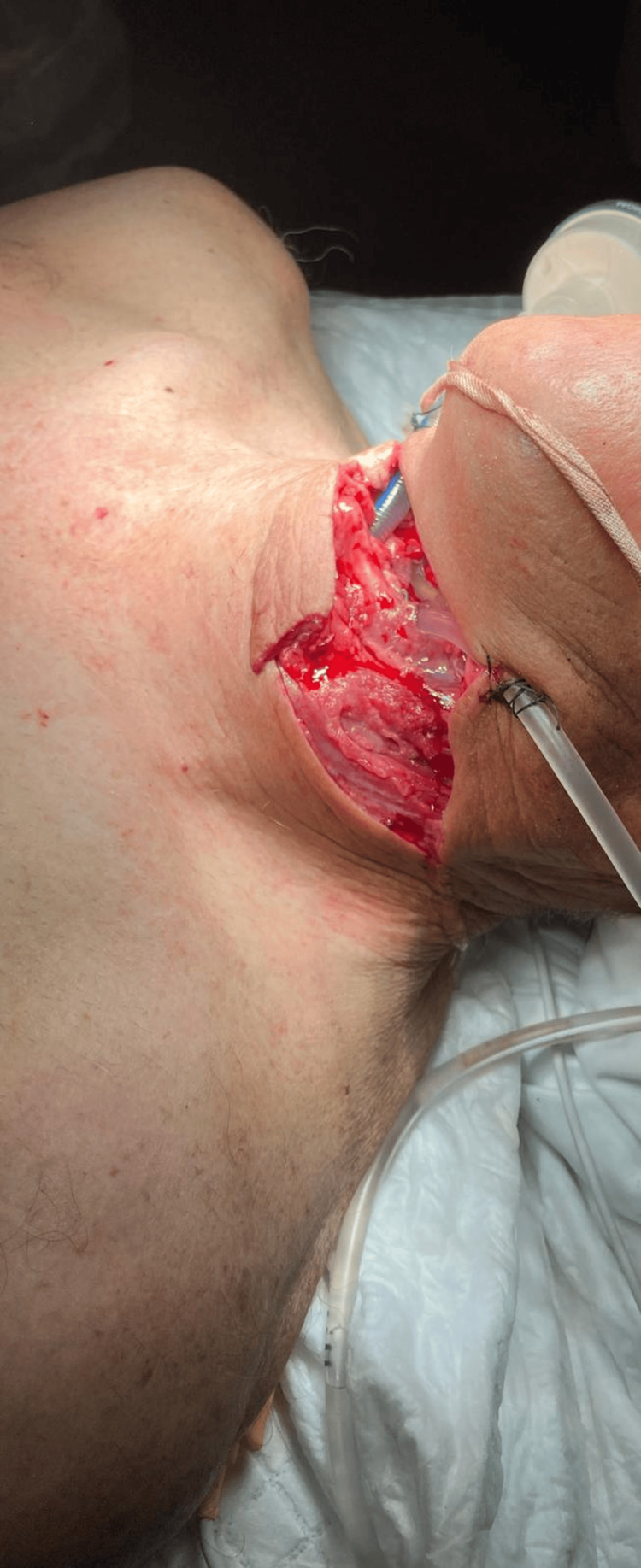
Self-inflicted cervical trauma showing an endotracheal tube positioned on the patient’s right side and a gastric tube positioned on the patient’s left side. Written informed consent was obtained from the patient for publication of their images in the journal.

**Figure 2:**
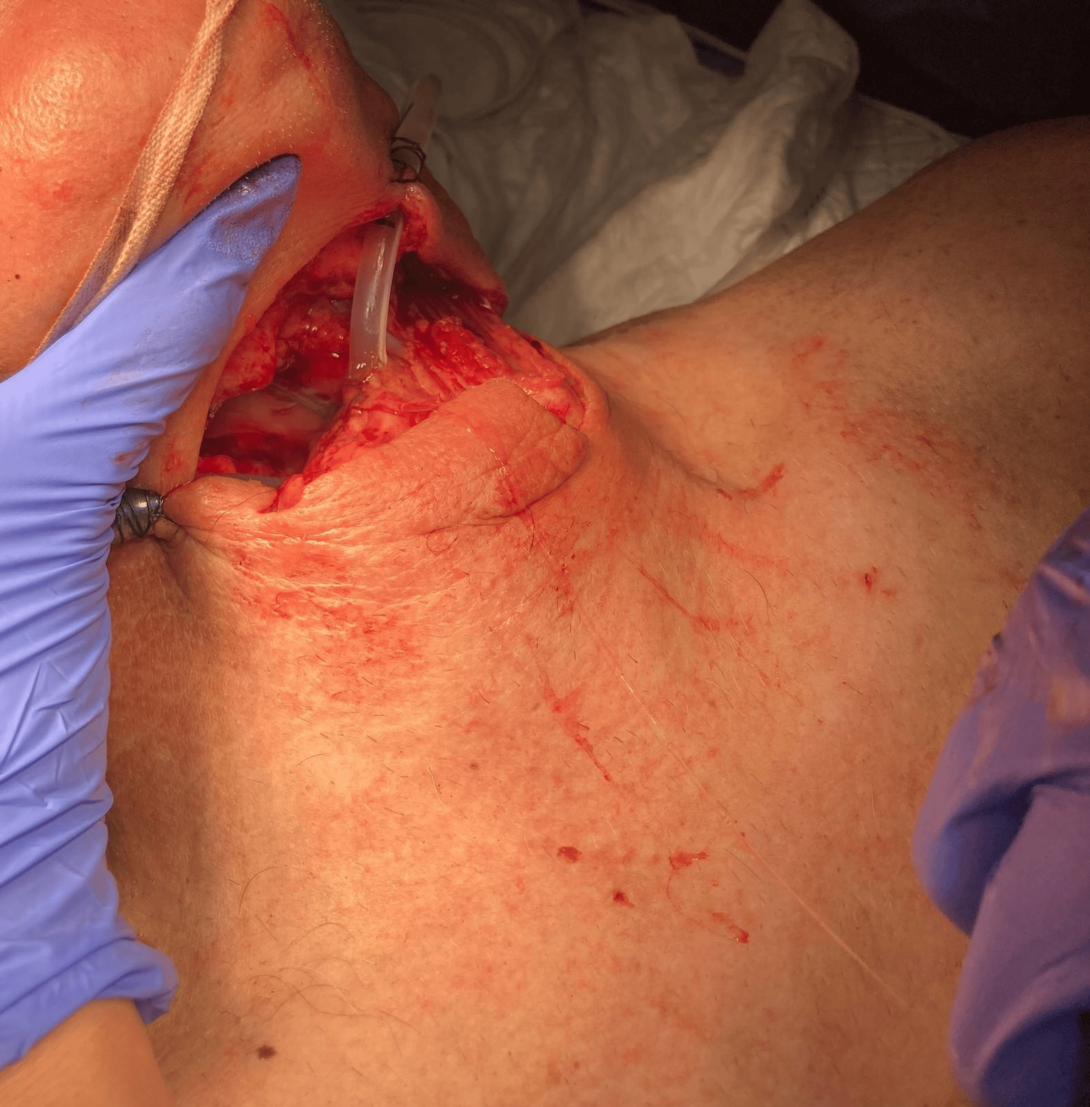
Transverse anterior cervical wound showing laryngeal (supraglottic) and pharyngeal transection. Written informed consent was obtained from the patient for publication of their images in the journal.

Immediately after the incident, he was transported by the pre-hospital medical emergency (comprising a physician and a nurse) to the nearest tertiary hospital, under spontaneous ventilation with a high-flow face mask, with a Glasgow Coma Scale score of 15 and without any sedation. Once in the emergency room, the patient was evaluated by an anesthesiologist and a surgeon and it was decided to secure the airway through the open wound. The patient was preoxygenated with high-flow face mask, sedated with ketamine, propofol and fentanyl and then a 6.5 mm wire-reinforced tube was cautiously advanced through the open wound and vocal cords (Figures 1-2) without complications. Correct tube placement was confirmed by direct visualization of the tube through de vocal cords, auscultation and capnography, followed by fixation to the skin with sutures (Figures 1-2). A gastric tube was inserted through the pharyngeal wound (Figures 1-2), a left chest drain was placed for pneumothorax drainage, and the cervical wound was temporarily packed with gauze. A single dose of esomeprazole and piperacillin-tazobactam was administered.

Then the patient was transferred to the nearest trauma center, where the patient was assessed by a multidisciplinary team including anesthesiology, intensive care, general surgery, and otorhinolaryngology. On arrival at the emergency room, the following ABCDE assessment was performed: A (Airway): The airway was secured with a 6.5 mm wire-reinforced endotracheal tube. The cervical injury showed no active bleeding; B (Breathing): The patient was on mechanical ventilation, with a tidal volume (Vt) of 500 mL at 16 bpm, positive end-expiratory pressure (PEEP) of 6 cmH_2_O, and an inspired oxygen fraction (FiO_2_) of 30%. Minute ventilation was 7.4 L/min, with a peripheral oxygen saturation (SpO_2_) of 99%. The chest tube was bubbling but not draining. Pulmonary auscultation revealed decreased breath sounds on the left hemithorax, with rhonchi and transmitted sounds; C (Circulation): Blood pressure was 128/91 mmHg and heart rate 79 beats per minute (bpm), with no vasopressor support. Heart sounds were regular, with no murmurs; D (Disability Neurology): The patient was sedated with propofol 2% and received analgesia with fentanyl. The Richmond Agitation-Sedation Scale (RASS) score was -4. Pupils were 2 mm, equal, and reactive. Blood glucose was 143 mg/dL; E (Exposure/Environment): Cervical and thoracic injuries appeared stable, with no active bleeding. The abdomen was soft and non-tender, with no peritoneal signs. No additional trauma-related skin lesions were identified.

Arterial blood gas analysis showed a pH of 7.381, a partial pressure of carbon dioxide (pCO_2_) of 38 mmHg, and a partial pressure of oxygen (pO_2_) of 135.8 mmHg, as shown in Table 1. Laboratory studies revealed an Hb 13.2 g/dL and a lactate 0.78 mmol/L. CT imaging confirmed extensive subcutaneous emphysema, left pneumothorax, and left lower lobe atelectasis.

**Table 1:** Arterial blood gas and electrolyte values at admission to the emergency room of a level I trauma center, obtained while the patient was on mechanical ventilation with an FiO₂ of 30%. PaCO_2_: partial pressure of carbon dioxide; PaO_2_: partial pressure of oxygen; HCO_3_⁻: bicarbonate; SaO_2_: arterial oxygen saturation; Na^+^: sodium; K^+^: potassium

Parameter	Value
pH	7.381
PaCO_2_ (mmHg)	38
PaO_2_ (mmHg)	135.8
HCO_3_⁻ (mmol/L)	22.0
SaO_2_ (%)	98.9
Na^+^ (mmol/L)	136.4
K^+^ (mmol/L)	3.69

Considering the patient’s overall clinical presentation, he was classified as experiencing class I hemorrhagic shock, with no need for blood transfusion.

**Figure 3:**
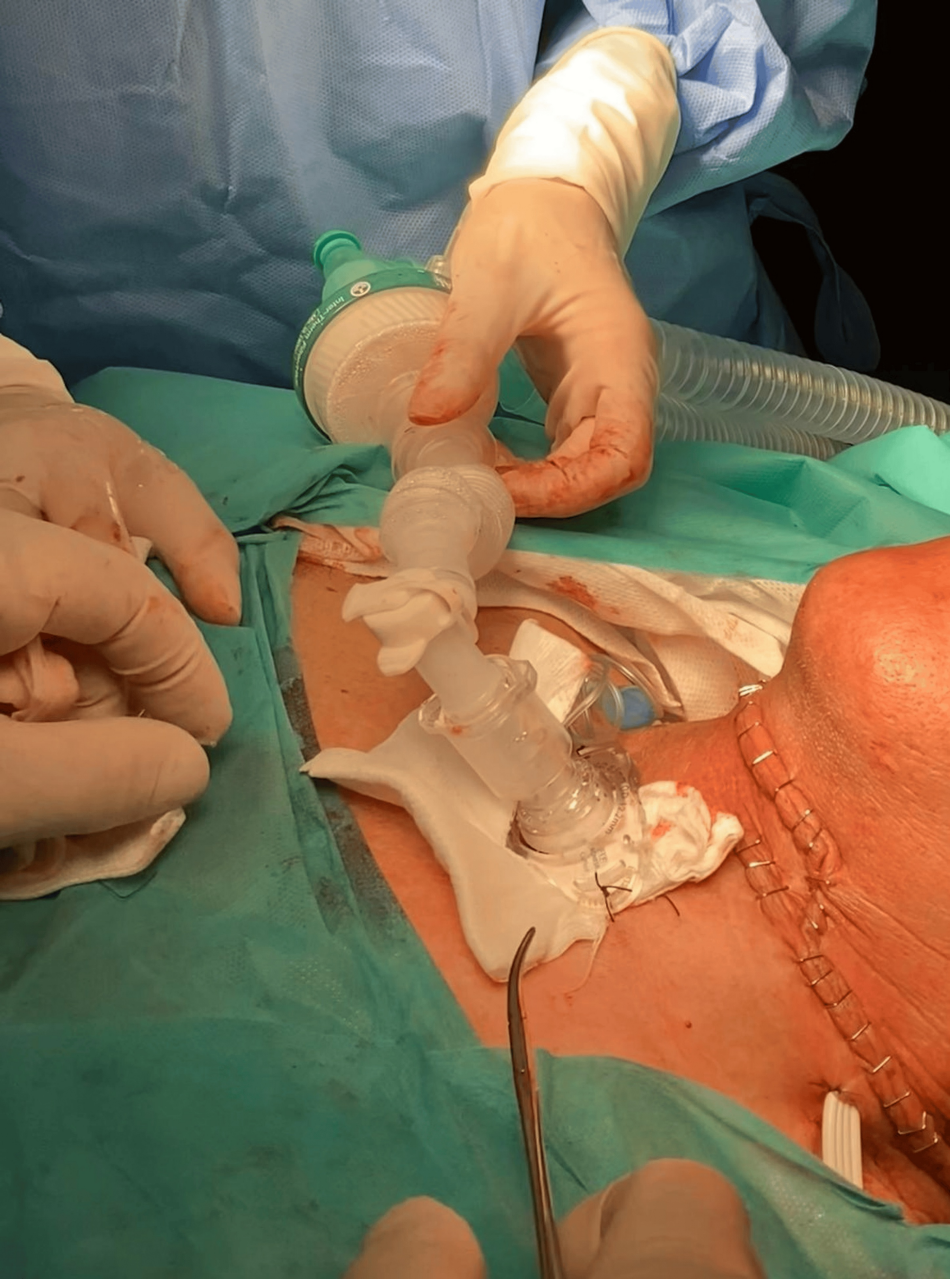
Postoperative appearance of the tracheostomy and anterior neck exploration site following pharyngeal reconstruction. Written informed consent was obtained from the patient for publication of their images in the journal.

The patient was transported to the operating room for tracheotomy and layered pharyngeal reconstruction under general anesthesia (Figure 3). During the procedure, the patient was monitored according to ASA standards with the addition of invasive blood pressure monitoring, maintained on mechanical ventilation in volume-controlled mode (Vt 5mL/kg, respiratory rate (RR) 12-16 bpm, PEEP 5 cmH_2_O, FiO_2_ 40%), under intravenous general anesthesia with propofol, fentanyl, and rocuronium. Serial arterial blood analyses were performed, and based on the patient’s hemodynamic status, blood transfusion was deemed unnecessary; management consisted of fluid resuscitation along with a low-dose norepinephrine infusion. The patient sustained an open cervical wound at the thyrohyoid membrane with epiglottis transection and pharyngeal exposure. A tracheostomy was performed, the pharyngeal laceration was closed in layers, and the inferior edge of the epiglottis was reattached to the thyroid cartilage. Esophageal integrity and cervical neurovascular bundles were preserved, and a nasogastric tube was repositioned and secured.

Postoperatively, the patient was admitted to the ICU, where he remained hemodynamically stable. He remained on mechanical ventilation and was transitioned to spontaneous ventilation via tracheostomy on postoperative day 2. Sedation with propofol and fentanyl was gradually transitioned to dexmedetomidine and subsequently weaned. On postoperative day 7, the patient was transferred from the ICU to the otorhinolaryngology ward. An increase in C-reactive protein and episodes of diarrhea were observed, leading to a diagnosis of Clostridium difficile infection, and antibiotic therapy was switched to vancomycin, with both clinical and analytical improvement within two days. On day 8, he was successfully extubated and maintained on spontaneous breathing through the tracheostomy. On postoperative day 10, the patient received psychiatric evaluation and management. Further follow-up was not possible, as the patient was transferred to a prison healthcare facility for legal reasons.

## Discussion

As described by Loss et al., there are three traditional zones of the neck: Zone I extends from the clavicles and sternal notch to the cricoid cartilage, Zone II is located between the cricoid cartilage and the angle of the mandible, and Zone III extends from the angle of the mandible to the base of the skull. Zone II, which corresponds to the injured area in this case, is particularly critical due to its high density of vascular structures, and catastrophic complications may occur in the absence of optimal management [1,2].

Patients with cervical and thoracic injuries often present with a predictable difficult airway, compromised ventilation and oxygenation, and may also experience hypovolemic shock due to substantial hemorrhage, requiring a rapid and coordinated multidisciplinary approach [3].

Given that this was a trauma patient, he should be considered as having a full stomach, and there are no data regarding whether rapid sequence induction (RSI) or appropriate prophylaxis for aspiration was implemented. These factors underscore the heightened risk of airway complications in trauma settings [5,6].

Patients with penetrating neck trauma often present with complex airway challenges; distorted anatomy, bleeding, and hypoxia may render conventional airway management impossible. According to the current Difficult Airway Society guidelines, awake fibreoptic or video-assisted intubation is recommended in situations where a predictably difficult airway is anticipated-such as expected difficulty with laryngoscopy, mask ventilation, supraglottic device placement, or front-of-neck access. These techniques are particularly indicated when loss of airway control after induction would be hazardous, as they allow maintenance of spontaneous ventilation and controlled progression toward securing the airway. In such settings, sedative agents like ketamine or carefully titrated propofol are preferred because they facilitate patient cooperation while preserving respiratory drive [5-7]. However, these awake approaches are only feasible when airway continuity is preserved and adequate visualisation is possible. In cases of complete or near-complete laryngotracheal transection-such as in our patient-these conditions are absent, making awake fibreoptic or video-assisted intubation impossible. Under such circumstances, emergent surgical airway access or direct intubation through the open wound becomes the only viable life-saving strategy [8-11].

Conversely, as described in the literature, alternative airway management strategies, such as fiberoptic-assisted intubation or the use of adjuncts, including a bougie or frova introducer, could be considered in selected cases [2,8-9]. Nevertheless, the initial airway management was conducted by another team at a non-tertiary hospital, where material resources are often limited. It is important to note that direct intubation through the site of airway transection is associated with potential complications, including wound infection, tracheal stenosis, vocal cord injury, and intraluminal trauma [8,11]. These risks underscore the importance of meticulous planning and follow-up, particularly when unconventional airway interventions are required in trauma patients [11].

The role of the anesthesiologist is crucial in the management of complex airway injuries. However, in hospitals without anesthesiology services, it is essential that intensivists, surgeons, and other critical care physicians are trained and prepared to manage cases with compromised airways. Beyond technical expertise, successful outcomes rely heavily on non-technical skills such as communication, teamwork, and shared understanding of the situation. Effective coordination between anesthesiology, surgery, and critical care teams ensured timely decision-making and optimal patient outcomes [1].

Another key consideration is the psychiatric dimension of self-inflicted neck injuries. These patients often present after impulsive or severe suicidal behavior, and multidisciplinary care should ideally include early psychiatric assessment and support once the acute phase is stabilized [8,9]. Unfortunately, in this case, further follow-up was unavailable after transfer to a correctional healthcare facility.

## Conclusions

Cervical trauma with complete airway transection demands rapid, innovative, and coordinated multidisciplinary management. Effective airway management through unconventional approaches requires a multidisciplinary team to ensure both airway control and overall patient stability. This case highlights the importance of early recognition, effective teamwork, and adaptable airway strategies in managing complex neck injuries.
